# Use of irradiated chitosan as a matrix for slow-release urea and *in vitro* fermentation characteristics of slow-release urea supplementation in ruminant rations

**DOI:** 10.14202/vetworld.2024.319-328

**Published:** 2024-02-07

**Authors:** Wahidin Teguh Sasongko, Teguh Wahyono, Dewi Apri Astuti, Akhmad Rasyid Syahputra, Slamet Widodo, Anuraga Jayanegara

**Affiliations:** 1 Graduate School of Nutrition and Feed Science, IPB University, Bogor 16680, Indonesia; 2 Research Center for Animal Husbandry, National Research and Innovation Agency of Indonesia, Bogor 16911, Indonesia; 3 Research Center for Food Technology and Processing, National Research and Innovation Agency of Indonesia, Gunungkidul 55861, Indonesia; 4 Department of Nutrition and Feed Technology, Faculty of Animal Science, IPB University, Bogor 16680, Indonesia; 5 Research Center for Radiation Process Technology, National Research and Innovation Agency of Indonesia, South Tangerang 15314, Indonesia

**Keywords:** fermentation characteristics, irradiated chitosan, ruminant, slow-release urea

## Abstract

**Background and Aim:**

Irradiated chitosan can be used as a matrix for slow-release urea (SRU) production. This study aimed to (1) determine the optimal formulation of irradiated chitosan matrix for controlling nitrogen release and (2) evaluate the characteristics of SRU *in vitro* fermentation based on irradiated chitosan as a feed supplement.

**Materials and Methods:**

In the first phase of the investigation, four chitosan-based SRU formulations with varying amounts of acrylamide (3 and 5 g) and gamma irradiation (5 and 10 kGy) were evaluated. Scanning electron microscopy, Fourier transform mid-infrared spectroscopy, and ammonia release characteristics were used to observe morphological, functional group, and ammonia release characteristics. In the second phase of research, the most effective SRU formulation was utilized as a supplement to ruminant rations based on rice straw, sorghum straw, and alfalfa. Gas production, rumen fermentation characteristics, and methane gas production were observed *in vitro*.

**Results:**

On the basis of surface image analysis, the four SRU formulas generate a similar appearance. Compared with untreated urea, the SRU3 formula reduced the percentage of ammonia emission by 12.85%–27.64% after 24 h of incubation (p = 0.05), as determined by the first phase study. SRU3 became the basis for the second testing phase. The addition of SRU3 did not affect the optimal gas production *in vitro*. SRU3 treatment produced less gas than Optigen® treatment (p = 0.05). With regard to rumen fermentation and digestibility, Optigen® yielded better results than SRU3 (p = 0.05). However, the treatment with SRU3 resulted in reduced methane production compared to that in the control (p = 0.05).

**Conclusion:**

Irradiated chitosan as an SRU matrix may control the release of ammonia in the rumen medium. The SRU3 formulation is the most effective. The addition of SRU to rice straw-based rations reduces methane production without affecting *in vitro* digestibility.

## Introduction

Indonesia has one of the longest coastlines in the world [1]. This is an opportunity in the form of an available natural resource consisting of crustacean shells. Raw materials, such as crab shells, shrimp shells, and lobster shells, can be extracted into chitosan with a higher functional value. Chitosan is a chitin polysaccharide and deacetylated polymer. Chitosan, a marine-derived natural product, exhibits antibacterial activity, biodegradability, and biocompatibility, which makes it useful in agriculture, medicine, food processing, and biotechnology [2, 3]. Amino, hydroxy, and oxygen bridge functional groups located at C-2, C-3, and C-6 positions are responsible for the biological activities of chitosan molecules [4]. Chitosan is a prospective biomaterial for use in nutrition, health, and animal husbandry due to the unexpected global increase in the price of purchased feeds and medicine, as well as the precipitous decrease in livestock productivity [5].

A recent meta-analysis [6] suggested that chitosan may be a natural rumen modulator. This is due to its ability to positively affect rumen fermentation, particularly by improving propionate levels and decreasing acetate levels. These changes are beneficial because they stimulate higher energy synthesis and possibly reduce methane emissions. Chitosan changed fermentation through metabolically more efficient pathways without decreasing organic matter digestibility, but it tended to decrease neutral detergent fiber digestibility and ammonia concentration [7]. Chitosan supplementation does not negatively affect feeding efficiency, rumen fermentation, milk production, or milk quality [8]. The biological, chemical, and physical properties of chitosan are evaluated based on the molecular weight and degree of deacetylation [9]. Low-molecular-weight chitosan is the most effective type of chitosan used in the livestock industry [5].

Chitosan has been successfully incorporated into slow-release urea (SRU) materials due to its biological activity and degree of acetylation. Chitosan has the potential to be used as a physical barrier that effectively minimizes the rate of water diffusion into the central nitrogen core [10, 11] in SRU for fertilizer application. The addition of SRU to a ration containing a protein source protected by condensed tannin does not affect the activity of microbial protein synthesis in the rumen and enhances post-ruminal protein availability [12]. Gamma irradiation treatment can enhance the beneficial effects of chitosan as a constituent material for SRU. With gamma irradiation, the viscosity and molecular weight of chitosan decrease in a dose-dependent manner [13]. This decrease is due to the depolymerization of chitosan chain through scission mechanism [14]. However, the characteristics of irradiated chitosan need to be evaluated to determine its effectiveness as a ruminant feed. Salami *et al*. [15] investigated SRU products made from urea identically coated with semi-permeable vegetable fat.

To the best of our knowledge, no information is available on the use of irradiated chitosan as an SRU ingredient in feed supplements. Therefore, this study was designed to evaluate (1) the optimal formulation of irradiated chitosan matrix for controlling nitrogen release in the rumen and (2) the characteristics of *in vitro* fermentation of SRU based on irradiated chitosan as a feed supplement for ruminants.

## Materials and Methods

### Ethical approval

This study used rumen fluid as a medium for the *in vitro* experiments. Nevertheless, ethical approval was not required for this study since the inoculum was obtained from slaughterhouse waste.

### Study period and location

This study was conducted from December 2022 to May 2023 at Research Center for Radiation Process Technology, National Research and Innovation Agency of Indonesia, South Tangerang, Indonesia.

### Experiment 1

#### SRU preparation

Four SRU formulations were investigated in this study. Table 1 shows the composition of the SRU material. Polyvinyl alcohol, acrylamide, and demineralized water were obtained from the Research Center for Radiation Process Technology of the Radiation Process Laboratory, Research Center for Radiation Process Technology. Maize starch was purchased from traditional markets. Chitosan solution was obtained from shrimp shell materials extracted from North Jakarta, Indonesia. Chitosan was irradiated at a dose of 75 kGy before inclusion in the mixture formulation. Irradiation was performed using a Karet Alam Gamma Irradiator (IRKA; Kimura Ltd., Japan). Polyvinyl alcohol, acrylamide, irradiated chitosan, and demineralized water were blended at 11× *g* and 90°C for 20 min. Subsequently, the prepared solution was wrapped in plastic and radiated at a dose of 5 or 10 kGy (Gamma cell, dose rate of 2.8 kGy). The ratio of formula to urea in the mixture formulation is 1:7. The SRU formulation is compressed into pellets.

**Table-1:** Ingredients of irradiated chitosan matrix.

Sample	Maize starch (g)	Acrylamide (g)	Poly vinyl alcohol (g)	Chitosan (ml)	Irradiation dosage (kGy)
SRU1	5	3	3	5	5
SRU2	5	5	3	5	5
SRU3	5	3	3	5	10
SRU4	5	5	3	5	10

SRU=Slow-release urea

#### Experimental design

Four different SRU formulations (Table-1) were evaluated using a completely random design. Each treatment was repeated 4 times.

#### Scanning electron microscopy (SEM) measurement

SRU surface area characteristics were evaluated by SEM. The sample was placed in a gold- and palladium-coated plate that served as a conductor before observation. Observations were performed at an accelerated energy of 15 kV. We compared and examined the derived images at 500× and 1000× magnification in detail.

#### Fourier transform mid-infrared spectroscopy (FTIR) measurement

2 mg sample and 200 mg K Br were quickly and homogeneously mixed in a mortar to produce the preparation sample. The sample was analyzed in 60 s using an IRPrestige-21 FTIR spectrometer (Shimadzu, Japan) in the 4000-500 cm^−1^ wave range. Peak positions were identified using Shimadzu IR solution 1.50 (Shimadzu).

#### In vitro fermentation

Rumen fluid was obtained from two freshly slaughtered (approximately 300 kg live weight) cattle at an abattoir in South Tangerang, Banten, Indonesia, using the method of Wahyono *et al*. [16] with some modifications. Rumen fluid received modifications by pre-use incubation in a water bath at 39^o^C and using a fermentation gas production flow pipe. We created buffers based on those described by Menke *et al*. [17]. SRU samples (5 g) were incubated in 30 mL of rumen fluid buffer medium (1:2 v/v). Incubation at 39°C for 24 h was performed. Ammonia release and pH measurements were performed at 3, 6, 9, and 24 h. The incubated samples were centrifuged at 504× *g* for 10 min before measurement.

#### Ammonia release and pH determination

The release of ammonia was analyzed according to the Conway procedure [18]. We measured the pH values using a pH meter (Hanna Instrument®, Rhode Island, USA). For Experiment 2, we used the composition and pH of SRU with stable ammonia release.

### Experiment 2

#### Ruminant ration preparation for in vitro fermentation

In Experiment 2, a combination diet containing forage and concentrate with a dry matter (DM) ratio of 70% forage to 30% concentrate was used. The concentrate used for this study originated from a commercial concentrate containing a mixture of palm kernel meal, rice bran, cassava dregs, and coffee shell. Table-2 [19, 20] shows the nutrient profiles of the concentrate and forages. Rice and sorghum straw were obtained from the laboratory field in G.A Siwabessy Science and Technology Area, South Jakarta, Indonesia. Alfalfa is obtained from a fiber check sample from Ankom. Rice straw and sorghum straw were dried in an oven at an average temperature of 60°C for 72 h. Grinding is carried out until the sample exceeds 1 mm in size.

**Table-2:** Nutrient profiles of feed sample.

Nutrient content (% dry matter)	Feed sample			

	Rice straw	Sorghum straw	Alfalfa	Concentrate
Organic matter	78.43	89.77	87.72*	84.33
Ash	21.57	10.23	12.28*	15.67
Crude protein	6.55	7.25	15.30*	19.54
Crude fiber	-	-	25.91**	26.81
NDF	69.24	73.01	32.32**	-
ADF	44.06	47.74	28.10**	-

* Suwignyo *et al.* [19];

** ANKOM Fiber check sample [20], NDF=Neutral detergent fiber, ADF=Acid detergent fiber

#### Experimental design

Three different ruminant rations and four supplementation treatments were evaluated using a 3 × 4 factorial arrangement. Each treatment was repeated 3 times. The first factor is the difference in forage source (rice straw, sorghum straw, and alfalfa), while the second factor is supplementation (control, urea, SRU, and Optigen® [Alltech, Inc., Fukuoka, Japan]). The urea used in this study was analysis grade urea (Merck, Inc., USA). A commercial blended urea product known as Optigen® II (Alltech, Inc.) coated with vegetable oil was used in the experiments. Feed supplementation dosage is 1% DM from total rations per sample.

#### In vitro fermentation measurement

*In vitro* fermentation was determined using the *in vitro* gas method developed by Menke *et al*. [17]. Rumen liquid was collected from two freshly slaughtered cattle (approximately 300 kg live weight) at an abattoir in South Tangerang, Banten, Indonesia. Cattle were fed native grass mixed with 2 kg/d concentrate made from rice bran and cassava dregs. The buffer solution was prepared according to the method of Menke and Steingass [21]. Three replicates of 200 mg ration samples were weighed in a calibrated 100 mL syringe glass (Fortuna®, Labortechnik, Germany). As controls, three syringes with only rumen-buffer solution were incubated. Each syringe was incubated in a water bath at 39°C with 30 mL of rumen-buffer fluid. After incubation, gas production was measured at 3, 6, 9, 12, 24, and 48 h. Using non-linear regression and Statistical Package for the Social Sciences (SPSS) 25.0 software (IBM SPSS Statistics, Armonk, New York, USA), the kinetics of gas production were calculated using the equation of Ørskov and McDonald [22] as follows:

*p* = *a* + *b* (1-*e*^−*ct*^)

where p is the accumulated gas production (mL), t is the incubation time (h), a is the gas production obtained from the soluble fraction (mL/200 mg DM), b is the gas production obtained from the insoluble fraction (mL/200 mg DM), and c is the gas production rate (mL/h).

After 48 h of incubation, 20 mL of rumen-buffer was collected from representative samples to measure pH, ammonia (mg%, Conway 1951), and short-chain fatty acids (SCFAs) (mM) [23]. Methane production was estimated on the basis of SCFAs using the following equation [24]:

CH_4_ (mM) = (0.5 × acetate concentration) + (0.5 × butyrate concentration) – (0.25 × propionate concentration)

### Statistical analysis

SPSS version 25.0 (IBM Corp., Armonk, NY, USA) was used for statistical and one-way analysis of variance (ANOVA) analysis. The means of each treatment were compared using Duncan’s multiple range test. General linear model method was used to evaluate the interaction studies between forage sources in rations and feed supplementation.

## Results

### Characteristics of SRU based on irradiated chitosan

**Figure-1:**
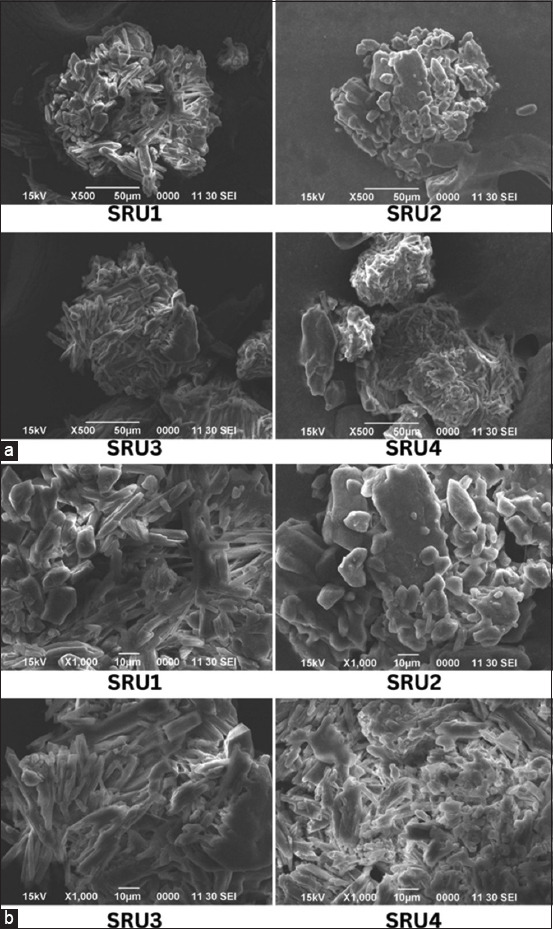
Scanning electron microscopy of four formulations of slow-release urea based on irradiated chitosan at (a) 500× and (b) 1000× magnification.

Figure-1 shows the surface image characteristics of the four SRU formulations analyzed by SEM. Figures-1a and b illustrate the 500× and 1000× SEM images, respectively. All four formulations presented a smooth and uniform surface. SRU3 and SRU4, which resulted from 10 kGy of irradiation, had surface conditions that appeared to be microscopic flakes in greater detail than SRU1 and SRU2.

**Figure-2:**
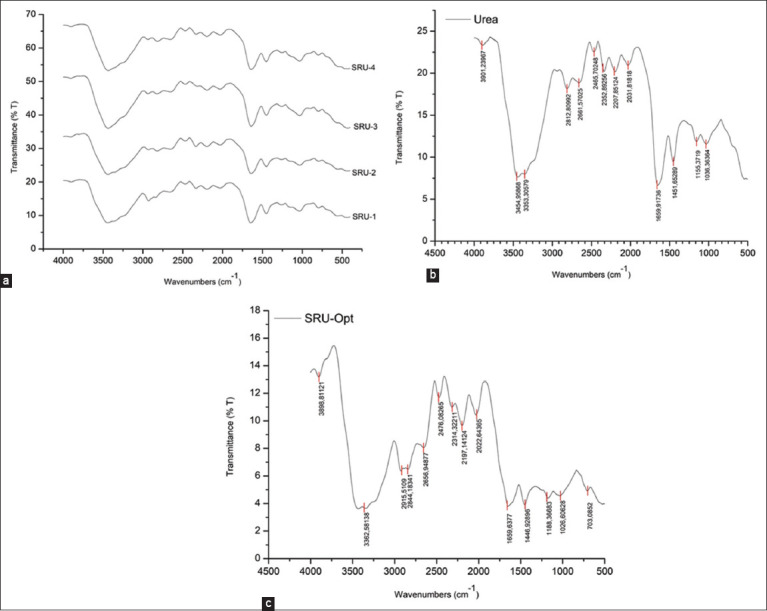
Fourier transform mid-infrared spectra of (a) four formulations of slow-release urea based on irradiated chitosan, (b) urea, and (c) optigen®.

Fourier transform near-infrared spectroscopy (Shimadzu) was used to characterize SRU, untreated urea, and Optigen (Figure-2). Figure-2a shows the Raman spectra of the four SRU formulas with the following results. Stretching vibrations in the H_2_O and N-H groups are indicated by the absorption bands at 3449.49, 3445.45, 3436.36, and 3440.91 cm^−1^ for SRU1-SRU4, respectively. For SRU1-SRU2, the stretching vibrations of CH_2_ and CH_3_ were observed at 2927.27, 2940.91, 2936.36, and 2940.91 cm^−1^, respectively. The absorption of urea (string) amide I at a wavenumber of 1636.36–1640.91 cm^−1^, which corresponds to the C–N stretching absorption of the open-chain compound NH_2_ with its helical structure, is also predictive of SRU attributes. A peak at 1026.59 cm^−1^ indicates the vibrational bending of the C–O–C group. At wave numbers of 3353.31, 1659.92, and 1451.65 cm^−1^, the -OH, -NH bend, and -CH groups were noticeable according to urea assays (Figure-2b). In Figure-2c (Optigen^®^), the -CH stretching, -NH, and C-O-C groups are observed at wave numbers 2844.18, 1659.64, and 1026.61 cm^−1^, respectively.

**Figure-3:**
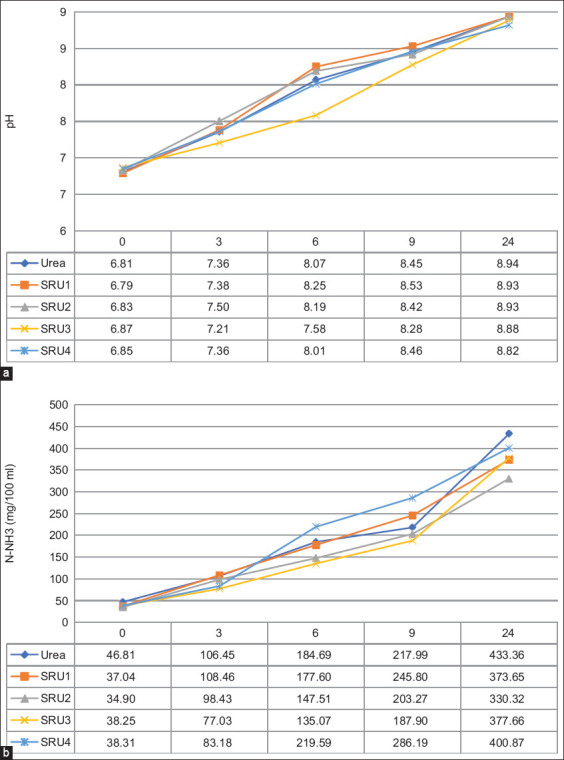
(a) pH value and (b) ammonia release of urea and slow-release urea based on irradiated chitosan by *in vitro* fermentation during 24 h of incubation.

Figure-3 presents (a) the pH value, and (b) the release of ammonia from four formulations of SRU based on irradiated chitosan after 24 h of *in vitro* fermentation. Based on the first phase *in vitro* test, the SRU3 formula produced a lower pH level than urea and other SRU treatments at 6 and 9 h of incubation (p < 0.05). There were statistically significant differences in pH parameters between urea and the four SRU formulations (p < 0.05). However, after 24 h of incubation, SRU4 produced the lowest pH, which was not significantly different from that of SRU3. After incubation at 0 h, the increase in ammonia due to sample incubation varies according to the release of N fermentation products. Compared with untreated urea, the SRU3 formula reduced ammonia release by 12.85% and 27.64% (p < 0.05). Neither SRU1 nor SRU4 exhibited significant differences with urea. SRU3 was used as a supplement in the following phase of the examination based on its ability to control ammonia release during the first phase.

### *In vitro* fermentation characteristics of SRU supplementation in ruminant rations

Table-3 presents the total and kinetics of gas production in ruminant rations supplemented with urea, Optigen®, and SRU3. Differences in forage significantly affected gas production *in vitro* during incubation for 3–48 h (p < 0.01). The effect of supplementation on potential gas production (a + b) was statistically significant (p < 0.05). The interaction between forage and feed supplement significantly influenced gas production but only during the 3^rd^ h of incubation (p < 0.05). Rice straw-based rations produced the lowest amount of total gas production (p < 0.05). It is interesting to note that supplement treatment (urea, SRU3, and Optigen®) did not significantly affect gas production, potential gas production (a + b), and gas production rate (c).

**Table-3:** *In vitro* gas production and kinetics of ruminant rations supplemented with urea, Optigen® and SRU3.

Rations	Supplement	Incubation time (h)						Gas kinetics	
	
		3	6	9	12	24	48	a+b	c
	
		mL/200 mg DM							
Rice straw+concentrate	Control	4.85^ab^	8.58^ab^	12.37^ab^	15.53^ab^	28.21^a^	41.02^a^	55.48^abc^	0.030^a^
	Urea	3.82^a^	7.02^a^	10.84^a^	14.17^a^	27.23^a^	40.42^a^	55.62^abc^	0.028^a^
	SRU3	3.83^a^	7.04^a^	10.86^a^	14.44^a^	27.03^a^	39.37^a^	52.31^a^	0.031^a^
	Optigen®	3.71^a^	7.05^a^	12.25^ab^	14.96^a^	28.07^a^	41.06^a^	53.89^ab^	0.031^a^
Sorghum straw+concentrate	Control	5.04^abc^	8.77^ab^	15.84^b^	15.34^ab^	25.41^a^	37.27^a^	63.65^f^	0.030^a^
	Urea	6.26^bc^	10.80^b^	15.34^b^	19.27^b^	32.15^a^	46.51^ab^	59.57^cdef^	0.032^a^
	SRU3	6.49^c^	10.77^b^	15.42^b^	19.34^b^	31.84^a^	46.78^ab^	60.97^def^	0.030^a^
	Optigen®	6.41^bc^	11.09^b^	15.04^b^	19.11^b^	32.67^a^	47.47^ab^	62.50^ef^	0.030^a^
Alfalfa+concentrate	Control	10.47^e^	19.45^c^	27.31^c^	32.67^c^	46.38^b^	56.35^b^	58.29^bcde^	0.069^b^
	Urea	10.01^de^	17.79^c^	25.69^c^	32.09^c^	43.84^b^	53.71^b^	55.18^abc^	0.070^b^
	SRU3	8.82^d^	18.68^c^	26.15^c^	31.30^c^	43.61^b^	53.72^b^	54.97^ab^	0.072^b^
	Optigen®	10.69^e^	20.15^c^	27.89^c^	33.05^c^	45.84^b^	56.04^b^	57.39^bcd^	0.070^b^
SEM		0.392	0.760	1.023	1.168	1.274	1.264	0.608	0.003
Forage		**	**	**	**	**	**	**	**
Supplement		ns	ns	ns	ns	ns	ns	*	ns
F*S		*	ns	ns	ns	ns	ns	ns	ns

The ration was composed of 70% forage and 30% concentrate by DM, the “control” group had no supplements, 3 mg of supplementation was given in each supplement treatment, potential gas production (a+b); gas production rate (c), different superscript in the same column means significant difference (p < 0.05);

* mean p *<* 0.05;

** mean p *<* 0.01, SRU=Slow-release urea, DM=Dry matter, SEM=Standard error mean

Table-4 presents the *in vitro* rumen fermentation and methane emission results for ruminant rations supplemented with urea, Optigen®, and SRU3. The addition of supplements to the diet did not affect pH and nC5 values. The addition of forage to the diet significantly affected the concentrations of ammonia, SCFA, C-2, and CH_4_ (p < 0.01). The effects of supplementation on ammonia, SCFA, C-2 (p < 0.01), C-3_,_ and CH_4_ (p < 0.05) levels were observed. Interactions were also observed between various forage sources and feed supplements in terms of SCFA parameters, C-2 and CH_4_ production (p < 0.01). N supplements significantly increased ammonia concentrations in all three ration types (p < 0.05). The addition of SRU3 to rice straw-based diet samples decreased SCFA and C-2 concentrations (p < 0.05). The treatment with Optigen® decreased SCFA and C-2 compared with alfalfa-based diet (p < 0.05). Regarding CH_4_ production, SRU3 may decrease its concentration in rice straw rations (p < 0.05). In addition, all alfalfa-based ration supplement treatments reduced CH_4_ concentrations (p < 0.05). The addition of supplements did not affect CH_4_ emissions from sorghum straw diets.

**Table-4:** *In vitro* rumen fermentation and methane emission of ruminant rations supplemented with urea, Optigen® and SRU3.

Rations	Supplement	pH	N-NH_3_	SCFA	C_2_	C_3_	iC_4_	nC_4_	iC_5_	nC_5_	CH_4_
		
			mg%	mM							mM
Rice straw + concentrate	Control	6.95^a^	36.41^ab^	123.35^f^	89.06^ef^	16.00^b^	1.79^b^	11.18^b^	2.96^ab^	2.35^a^	46.12^e^
	Urea	6.98^a^	39.72^cd^	118.07^ef^	88.29^ef^	12.51^ab^	1.53^ab^	10.11^ab^	3.27^ab^	2.35^a^	46.07^e^
	SRU3	6.95^a^	38.62^bc^	98.60^bcde^	67.39^bcd^	16.11^b^	1.37^ab^	9.08^ab^	2.94^ab^	1.71^a^	34.21^b^
	Optigen®	6.99^a^	41.72^de^	123.15^f^	90.04^f^	15.11^ab^	1.57^ab^	10.89^ab^	3.40^ab^	2.15^a^	46.69^e^
Sorghum straw +concentrate	Control	6.95^a^	34.10^a^	106.02^cdef^	73.20^cd^	14.89^ab^	1.51^ab^	10.46^ab^	3.43^ab^	2.53^a^	38.10^cd^
	Urea	6.96^a^	38.11^bc^	102.54^cde^	70.65^cd^	14.44^ab^	1.47^ab^	10.15^ab^	3.30^ab^	2.54^a^	36.79^cd^
	SRU3	6.94^a^	37.81^bc^	92.63^abc^	64.21^bc^	13.16^ab^	1.34^ab^	9.34^ab^	2.87^ab^	1.71^a^	33.48^bc^
	Optigen®	6.96^a^	37.91^bc^	100.98^cde^	66.90^bc^	15.66^ab^	1.53^ab^	11.11^b^	3.38^ab^	2.40^a^	35.09^bcd^
Alfalfa +concentrate	Control	6.95^a^	39.12^bcd^	115.17^def^	78.51^de^	16.94^b^	1.86^b^	11.44^b^	3.99^b^	2.43^a^	40.74^de^
	Urea	6.98^a^	43.73^e^	80.94^ab^	57.05^ab^	11.10^a^	1.18^a^	7.31^a^	2.61^a^	1.69^a^	29.41^ab^
	SRU3	6.94^a^	39.32^bcd^	95.41^bcd^	66.50^bc^	13.30^ab^	1.45^ab^	8.63^ab^	3.59^ab^	1.94^a^	34.24^bc^
	Optigen®	6.93^a^	43.93^e^	75.18^a^	47.31^a^	13.03^ab^	1.47^ab^	8.68^ab^	3.23^ab^	1.47^a^	24.74^a^
SEM		0.040	0.660	2.908	2.290	0.439	0.049	0.327	0.107	0.127	1.198
Forage		ns	**	**	**	ns	ns	ns	ns	ns	**
Supplement		ns	**	**	**	*	ns	ns	ns	ns	*
F*S		ns	ns	**	**	ns	ns	ns	ns	ns	**

The ration was composed of 70% forage and 30% concentrate by DM, the “control” group had no supplements, 3 mg of supplementation was given in each supplement treatment, ammonia (N-NH_3_), short chain fatty acids (SCFA), acetate (C_2_), propionate (C_3_), iso butyrate (iC_4_), n butyrate (nC_4_), iso valerate (iC_5_), n valerate (nC_5_), different superscript in the same column means significant difference (p < 0.05);

* mean p *<* 0.05;

** mean p *<* 0.01, SEM=Scanning electron microscopy, SRU=Slow-release urea, DM=Dry matter

## Discussion

### Characteristics of SRU based on irradiated chitosan

SEM was used to evaluate the morphological and microstructural properties of many types of SRU formulations. Urea coated with maize starch, acrylamide, polyvinyl alcohol, and chitosan exhibited a strong and consistent structure (Figure-1). The bottom pores of each of the four SRU formulas are similar. These results suggest that cross-links exist inside the membranes [25]. Jayanudin *et al*. [26] also found similar results, demonstrating that chitosan microspheres exhibited acceptable sphericity and smooth surface despite inconsistent diameter. Chitosan microspheres have a great spherical morphology with shiny surfaces [27]. This similar appearance reflects the ratio of the chitosan matrix to urea at 1:7. Variations in the acrylamide concentration and irradiation dose did not affect the visual characteristics of SRU surfaces. Differences in the binding of the chitosan matrix to urea were difficult to observe. It requires high-resolution transmission electron microscopy is required [28].

To observe the interaction between urea and the basic components of the chitosan matrix, FTIR measurements of SRU are necessary. The FTIR results related to urea and Optigen were also examined to observe the differences. Notably, the wavenumbers associated with the C–N stretch exhibited a peak transition, particularly in SRU2 and SRU4. The main reason for this is the higher acrylamide content in SRU1 and SRU3. The peak wavenumber for SRU2 and SRU4 is 1640.91 cm^−1^, whereas that for SRU1 and SRU3 is 1636.36 cm^−1^. According to Jayanudin *et al*. [27], the peak observed at 1633 cm^−1^ can be attributed to the Schiff base, specifically the C=N stretching. However, the unconjugated C=N stretch was detected at 1653 cm^−1^. The CH_2_- and -CH_3_ vibrations display a similar form, as indicated by the peak transition from 2927.27 at SRU1 and SRU3 to 2940.91 at SRU2 and SRU4. Peak wave numbers 2927.27–2940.91 are unique to SRU containing acrylamide, whereas those of urea do not (Figure-2b). Murugan *et al*. [29] calculated symmetric and asymmetric CH_2_ stretching vibrations of 2958 and 2930 cm^−1^ in the A and E forms, respectively. The absence of several wavenumber peaks in our FTIR test indicates that chitin deacetylation during the chitosan manufacturing process is clearly noticeable. The decreased strength of the bands corresponding to NH groups (3256 cm^−1^) and secondary amide N–H bonds (1551 cm^−1^) is predictive of deacetylation degree in chitosan [3].

In the first phase of our study, the release of SRU, represented by variations in pH, was determined by observing the pH kinetics following *in vitro* rumen fermentation. According to Aschenbach *et al*. [30], the pH value at the beginning of incubation (0 h) was in the normal range. However, after the *in vitro* fermentation process, the pH value increased due to the increase in ammonia release. A higher pH value is associated with a higher concentration of ammonia after the addition of N [31]. Ammonia produced in the rumen may help to regulate the pH by removing NH_4_+ [32]. SRU3 was able to control the decrease in pH when compared to the other four treatments. It has been suggested that the efficacy of SRU3 in limiting pH elevation can be attributed to its control mechanism that delays ammonia production, not exclusively due to its chitosan composition. Harahap *et al*. [6] showed that the addition of chitosan to the diet of ruminants did not result in a significant change in the pH. Shah *et al*. [32] and Seankamsorn *et al*. [8] also confirmed similar findings. It should be noted that the sample did not contain any additional feed during the first phase; therefore, the pH value indicates the fermentation process regardless of the N supplement. Rumen microbes are unable to directly utilize ammonia as the primary product due to the absence of carbon (C) sources, with the notable exception of a minor amount of starch in the SRU. If an energy source is present in the ration, the pH will not be affected by the variation in N sources, whether encapsulated urea or urea [33].

Our initial hypothesis was that the chitosan matrix influences the amount of ammonia released in several of the observed SRU formulations. As illustrated by the kinetics in Figure-3b, SRU2 and SRU3 exhibit this ability. The ammonia production kinetics are consistent with the pH kinetics. Because these two substances are closely related, particularly for single SRU and urea samples, NUE and ammonia evaporation from urea are enhanced by the chitosan contained in SRU [26]. Urea degradation in the rumen can be minimized by coating the material with a chitosan-based biopolymer [34]. In addition, gamma irradiation improves chitosan effectiveness. When gamma irradiation is used, its molecular weight and viscosity decrease in dose-dependent trends [13]. Therefore, the protective effect of the urea element will be further enhanced. The chitosan used in this study was irradiated at a dose of 75 kGy. The molecular weight of chitosan decreases rapidly when treated with radiation doses ranging from 20 to 200 kGy. As a result, the degradation rate was low [35]. Following the investigation in the first phase, the SRU3 formula was chosen to slow down the release of ammonia from urea without adding rations.

### *In vitro* fermentation characteristics of SRU supplementation on ruminant rations

In the second study, the performance of SRU as an N supplement was compared with that of urea and Optigen® following the evaluation of an individual sample. In addition, we were interested in assessing the beneficial effects of this supplement in three ration compositions that represented varying quality levels: Low (rice straw), medium (sorghum straw), and high (alfalfa). *In* *vitro* gas production is a suitable method for assessing the actual use of N supplements. A benefit of this methodology is that the gas produced is the final outcome being measured, directly results from microbial metabolism [36]. N supplementation (urea, SRU3, and Optigen^®^) did not significantly affect *in vitro* gas production or production rates (c) in any of the three dietary types. This finding indicates two distinct facts: (1) the addition of N to rations containing energy sources improves the synchronization of fermentation by microbes, and (2) the incorporation of SRU3 has no negative impact on rumen microbial metabolism. N sources can be utilized more effectively when integrated into energy sources; in this case, synchronization increases the efficiency of the microbial ammonia conversion process in the form of glutamate, thereby minimizing nitrogen and energy losses [36]. The use of irradiated chitosan had no significant effect on the total gas production and the gas production rate (c). Sirakaya and Beyzi [37] and De Queiroz Vieira *et al*. [38] have reported similar findings. The forage concentration in the ration had a greater impact on the total gas production in the samples compared to the supplementation treatment. Conversely, Haryati *et al*. [39] discovered that chitosan supplementation can decrease cumulative gas production. This difference was due to the amount of chitosan used as a supplement.

In contrast to the findings presented in the first phase, there was no significant difference in pH between the SRU treatment and control as well as the other N source treatments. This may be because the ration contains an energy source. Similar findings were reported by De Queiroz Vieira *et al*. [38] and Nayohan *et al*. [34]. The pH level in the rumen is crucial for optimizing performance and stability by influencing the microbial population, rumen fermentation products, and regular physiological activity [32]. Although SRU contains chitosan components, the stable pH in this study (6.93–6.99) indicates that SRU does not adversely affect rumen fermentation. Chitosan supplementation does not negatively impact feed use or rumen fermentation [6, 8]. SRU3 successfully preserves the rumen microbial fermentation environment in diets of variable levels (low-high quality rations). In contrast, according to Inácio *et al*. [36], the provision of soluble carbohydrates may reduce ruminal pH. As a result, the amount of ammonia in the form of ammonium ions increases and the ruminal epithelium absorption decreases. These differences may be due to variations in the composition of the rations.

Because the three dietary supplements compared in the present study are N sources, the production of ammonia will be greater than that in the control group. The concentration of ammonia in the rumen increases linearly with the amount of N supplement [40]. Urea is an effective choice for enhancing rumen fermentation, nutrient absorption, and digestibility due to its low cost as a nitrogen supplement. Urea supplementation improves ammonia production, crude protein digestibility, N intake, and daily gain in sheep [31]. Ammonia concentration in the rumen is mostly affected by factors such as protein content in the rations, ruminal pH, and protein solubility of feed ingredients [34]. However, there was no statistically significant effect of SRU on ammonia levels in rice straw and alfalfa-based forage. The content of irradiated chitosan in SRU3 may influence this issue. The ammonia concentration in the rumen was unaffected by the addition of chitosan [6, 38]. From the perspective of N utilization, controlling the release of urea in SRU3 offers the advantage of synchronizing N fermentation with energy supplied from the ration. As confirmed by the reduced methane emissions of the SRU3 and Optigen® treatments, chitosan alters the mechanism of rumen fermentation by modifying the fermentation pattern toward a more effective pathway for energy circulation when included in the diet of ruminants [32].

Because chitosan inside SRU3 has antimicrobial properties, it will influence the concentration of SCFAs *in vitro*. Chitosan can be used as a methane-reducing agent because it can exert antibacterial effects and alter fermentation [41]. Cell lysis is attributed to the alteration in cellular permeability caused by the electronegative charges on the surfaces of microorganisms and polycationic chitosan (R-NH_3_+), which has been identified as the principal antimicrobial mechanism of action of chitosan [38]. Chitosan shows potential as a natural rumen modulator due to its ability to induce favorable rumen fermentation. For example, it can increase propionate production while diminishing acetate levels, enhance energy synthesis and reduce methane emissions [6]. Methane production can be reduced by increasing the propionic concentration and acetate-to-propionate ratio of chitosan by 2% of dry matter intake [8]. Moreover, Shah *et al*. [32] reported that chitosan had little effect on the overall production of volatile fatty acids but significantly altered the fermentation process by changing the concentration from acetate to propionate. It is interesting to note that SRU3 and Optigen® have a specific influence on each ration. The addition of SRU3 to rice straw diet samples resulted in a reduction in SCFA and C-2 levels (p < 0.05). In addition, the Optigen® treatment showed the same pattern on the alfalfa-based diet. Salami *et al*. [15] reported similar findings, concluding that Optigen® enhances the efficacy of nutrient utilization in rations and reduces methane emissions. On the basis of our findings, SRU3 is effective for N supplementation in rice straw-based low-quality forage ration.

## Conclusion

Irradiated chitosan acts as an SRU matrix that can control the release of ammonia from the rumen medium. On the basis of our findings, SRU3 was the most effective formulation. SRU supplementation in rice straw-based rations can reduce methane production without affecting *in vitro* digestibility. SRU3 could be used as an environmentally beneficial feed additive due to its ability to reduce methane content. This argument is supported by *in vitro* experiments, particularly experiments on ruminants fed on low-quality feed (rice straw). SRU3 improves the efficacy of using low-quality forage as a feed supplement.

## Authors’ Contributions

AJ, TW, and DAA: Conceptualization and Supervision. WTS, TW, ARS, and SW: Methodology and formal analysis. AJ and DAA: Validation. TW, DAA, AJ, SW, and ARS: Investigation and data curation. WTS and TW: Writing original draft, review, and editing. TW, WTS, SW and AJ: Revised and edited the manuscript. All authors have read, reviewed, and approved the final manuscript.
